# The prognostic and therapeutic implications of circulating tumor cell phenotype detection based on epithelial–mesenchymal transition markers in the first-line chemotherapy of HER2-negative metastatic breast cancer

**DOI:** 10.1186/s40880-018-0346-4

**Published:** 2019-01-03

**Authors:** Xiuwen Guan, Fei Ma, Chunxiao Li, Shiyang Wu, Shangying Hu, Jiefen Huang, Xiaoying Sun, Jiayu Wang, Yang Luo, Ruigang Cai, Ying Fan, Qiao Li, Shanshan Chen, Pin Zhang, Qing Li, Binghe Xu

**Affiliations:** 1 Department of Medical Oncology, National Cancer Center/National Clinical Research Center for Cancer/Cancer Hospital, Chinese Academy of Medical Sciences and Peking Union Medical College, No. 17, Panjiayuan Nanli, Chaoyang District, Beijing, 100021 P. R. China; 2 State Key Laboratory of Molecular Oncology, National Cancer Center/National Clinical Research Center for Cancer/Cancer Hospital, Chinese Academy of Medical Sciences and Peking Union Medical College, Beijing, 100021 P. R. China; 3 SurExam Bio-Tech, Guangzhou Technology Innovation Base, Science City, Guangzhou, 510000 Guangdong P. R. China; 4 Department of Cancer Epidemiology, National Cancer Center/National Clinical Research Center for Cancer/Cancer Hospital, Chinese Academy of Medical Sciences and Peking Union Medical College, Beijing, 100021 P. R. China; 5 Department of Medical Oncology, Huanxing Cancer Hospital, Beijing, 100021 P. R. China

**Keywords:** Breast cancer, Circulating tumor cells, Epithelial–mesenchymal transition, Prognosis, Therapeutic implication

## Abstract

**Background:**

Epithelial–mesenchymal transition (EMT) is implicated in the metastatic process and presents a challenge to epithelial cell adhesion molecule-based detection of circulating tumor cells (CTCs), which have been demonstrated to be a prognostic indicator in metastatic breast cancer. Although evidence has indicated that heterogeneity of CTCs based on EMT markers is associated with disease progression, no standard recommendations have been established for clinical practice. This study aimed to evaluate the prognostic significance of dynamic CTC detection based on EMT for metastatic breast cancer patients.

**Methods:**

We enrolled 108 human epidermal growth factor receptor 2-negative metastatic breast cancer patients from the prospective phase III CAMELLIA study and applied the CanPatrol CTC enrichment technique to identify CTC phenotypes (including epithelial CTCs, biphenotypic epithelial/mesenchymal CTCs, and mesenchymal CTCs) in peripheral blood samples. Receiver operating characteristic curve analyses of total CTC count and the proportion of mesenchymal CTCs for predicting the 1-year progression-free survival (PFS) rate were conducted to determine the optimal cut-off values, and Kaplan–Meier analysis and Cox proportional hazards regression analysis were performed to investigate the prognostic value of the cut-off values of both total CTC count and the proportion of mesenchymal CTCs in combination.

**Results:**

For predicting the 1-year PFS rate, the optimal cut-off value of total CTC count was 9.5 (Area under the curve [AUC] = 0.538, 95% confidence interval [CI] = 0.418–0.657), and that of the proportion of mesenchymal CTCs was 10.7% (AUC = 0.581, 95% CI = 0.463–0.699). We used the two cut-off values in combination to forecast PFS in which the total CTC count was equaled to or exceeded 10/5 mL with the proportion of mesenchymal CTCs surpassed 10.7%. Patients who met the combined criteria had significantly shorter median PFS than did those who did not meet the criteria (6.2 vs. 9.9 months, *P *=0.010). A nomogram was constructed based on the criteria and significant clinicopathological characteristics with a C-index of 0.613 (*P *= 0.010).

**Conclusions:**

The criteria, which combine the total CTC count and the proportion of mesenchymal CTCs, may be used to monitor therapeutic resistance and predict prognosis in patients with metastatic breast cancer.

*Trial registration* *ClinicalTrials.gov*. NCT01917279. Registered on 19 July 2013, https://clinicaltrials.gov/ct2/show/NCT01917279?term=NCT01917279&rank=1.

## Background

In the past decade, circulating tumor cells (CTCs), identified as the “seeds” of lethal metastasis [1], have been considered as a novel biomarker to monitor therapeutic efficacy and predict cancer prognosis in multiple studies [2–7], propelling the approval of using CTC count measured with the CellSearch system to “monitor breast cancer treatment and indicate its effectiveness” by the U.S. Food and Drug Administration. Additionally, a pooled analysis of 1944 eligible patients in 20 studies confirmed the independent prognostic value of CTC count in metastatic breast cancer as level-I evidence [8]. Due to the evidence supporting CTC detection as a noninvasive and straightforward approach to achieve longitudinal monitoring of the treatment response and improve the cancer prognostication system, CTC count is included as an adverse prognosis factor for patients with primary and metastatic breast cancer in the 8th edition of the American Joint Committee on Cancer (AJCC) cancer staging manual [9].

Epithelial–mesenchymal transition (EMT) plays a critical role in promoting migration and invasion of stationary tumor cells and results in down-regulated expression of epithelial markers, which might lead to false-negative findings with epithelial cell adhesion molecule (EpCAM)-based detection [10–15]. A previous study suggested that mesenchymal markers are highly enriched in CTCs, although rare primary tumor cells simultaneously expressed both mesenchymal and epithelial markers [16]. Moreover, evidence has indicated an association between mesenchymal CTCs and therapeutic outcome of metastatic breast cancer, and the predictive value of total CTC count alone is limited due to the heterogeneity of CTCs [16]. Therefore, it is necessary to identify the mesenchymal markers overexpressed in CTCs, which are up-regulated during EMT. Although during the past decade, molecular and genomic profiling of single CTCs expanded our understanding of the heterogeneity of CTCs, the application of genome sequencing of single CTCs in clinical practice has been restricted by the prohibitive cost and complexity associated with these analyses [17]. Moreover, the prognostic value of mesenchymal CTCs for real-time monitoring of therapeutic resistance has seldom been investigated, and no cut-off value, criterion, or recommendation for such monitoring has been issued to guide clinical practice in metastatic breast cancer.

Our research group developed the CanPatrol CTC enrichment technique to isolate and characterize CTC based on epithelial markers (EpCAM and Cytokeratin [CK]) and mesenchymal markers (Vimentin and Twist) so as to classify CTCs into three subpopulations, namely, epithelial CTCs (E + CTCs), biphenotypic epithelial/mesenchymal CTCs (E+/M+ CTCs), and mesenchymal CTCs (M + CTCs) [18]. The CTCs expressing mesenchymal markers are defined as EMT-CTCs, comprising E+/M+ CTCs and M + CTCs. We, therefore, investigated the heterogeneity of CTCs with respect to EMT markers and hormone receptor (HR) status in 28 metastatic breast cancer patients using the previously described CanPatrol CTC enrichment technique [19].

The present study aimed to evaluate the prognostic value of dynamic CTC detection based on EMT markers at different therapeutic time points during the first-line chemotherapy in patients with human epidermal growth factor receptor 2 (HER2)-negative metastatic breast cancer. In addition, we investigated the association between the distribution of CTC subpopulations and patient clinicopathological characteristics, as well as their variations in CTC phenotype during treatment.

## Patients and methods

### Study design

Eligible patients were defined as women with metastatic breast cancer who agreed to participate in the multi-institutional CAMELLIA study (registered on *ClinicalTrials.gov*, Identifier NCT01917279), which was a prospective, randomized, open-labeled phase III study to explore the efficacy and safety of metronomic chemotherapy with capecitabine versus intermittent capecitabine as the maintenance therapy following first-line chemotherapy with capecitabine plus docetaxel in women with HER2-negative metastatic breast cancer at 32 clinical centers in China.

Eligible patients received capecitabine (1000 mg/m^2^ twice daily on days 1–14, every 3 weeks) plus docetaxel (75 mg/m^2^ on day 1, every 3 weeks) for a maximum of 6 cycles or until disease progression, intolerable adverse events, or patient withdrawal occurred. Patients with stable disease or a partial or complete response after initial chemotherapy were randomized to receive the maintenance chemotherapy with capecitabine of either conventional or metronomic dosage [20, 21]. A prospective translational study with longitudinal CTC analyses every 6 weeks during the first-line chemotherapy between November 2013 and July 2017 was designed to determine the prognostic value of dynamic CTC phenotype detection based on EMT marker composition. The study was approved by the local ethical committee, and informed consent was signed by every patient before entering the clinical trial.

The clinicopathological characteristics of the patients with detectable CTCs in the peripheral blood samples, including age, position and number of metastatic sites, HR status, and previous endocrinotherapy after confirmed tumor relapse were collected. Tumor response was assessed in accordance with the Response Evaluation Criteria in Solid Tumors (RECIST) guidelines version 1.1. Patients were required to undergo computed tomography, or magnetic resonance imaging if indicated, before first-line chemotherapy and after every two cycles of chemotherapy to evaluate disease and survival statuses. Disease-free survival (DFS) was defined as the duration between the date of breast cancer diagnosis and the date of clinical relapse confirmed by imaging. Progression-free survival (PFS) was defined as the duration between the date of enrolment and the date of clinically observed disease progression (according to RECIST criteria v1.1). Meanwhile, blood samples were collected synchronously until disease progression for the detection of serum tumor markers carcinoembryonic antigen (CEA) and carbohydrate antigen 153 (CA153) to determine CTC phenotype.

### Isolation, classification, and enumeration of CTCs

As has recently been described in details [18], CTCs were isolated, classified, and counted using the CanPatrol CTC filtration system, which includes a filtration tube (SurExam, Guangzhou, Guangdong, China) containing a calibrated membrane with 8-μm diameter pores (SurExam), a manifold vacuum plate with valve settings (Millipore, Billerica, MA, USA), an E-Z 96 vacuum manifold (Omega, Norcross, GA, USA), and a vacuum pump (Auto Science, Tianjin, China). Prior to filtration, blood samples were treated with red blood cell lysis buffer (154 mmol/L NH_4_Cl, 10 mmol/L KHCO_3_, and 1 mmol/L ethylenediaminetetraacetic acid [EDTA] in deionized water; Sigma, St. Louis, MO, USA) to remove erythrocytes. Phosphate buffer saline (PBS) with 4% formaldehyde (Sigma) was subsequently applied to resuspend the remaining cells. The cell suspension was transferred to a filtration tube and pumped at 0.08 or more MPa to collect isolated CTCs on the membrane.

A multiplex RNA-in situ hybridization (RNA-ISH) assay based on branched DNA (bDNA) signal amplification was applied to classify and count CTCs. Four epithelial biomarkers (EpCAM and CK8/18/19), two mesenchymal biomarkers (Vimentin and Twist), and a leukocyte biomarker (CD45) were used to capture and characterize CTC subpopulations. A detailed RNA-ISH assay was performed as previously described [18]. Three types of fluorescently labeled probes were added and incubated with cells. The sequences of the capture probes and bDNA signal amplification probes had been previously published and were synthesized by Invitrogen (Shanghai, China) [18]. The cell nuclei were stained with 4,6-diamidino-2-phenylindole (DAPI, Sigma), and the cells were analyzed with an automatic Axio Imager Z2 fluorescence microscope (Zeiss, Carl Zeiss Meditec AG, Germany). The red and green fluorescence signals represent the expression of epithelial and mesenchymal biomarkers, respectively. The white fluorescent signals represent the expression of CD45.

### Statistical analyses

The clinicopathological characteristics of the recruited patients at baseline were described in percentages of categorical variables. The associations between the distribution of CTC subpopulations at baseline and clinicopathological characteristics (HR status, DFS, and previous endocrinotherapy after confirmed tumor relapse) of patients were analyzed using Mann–Whitney *U* test. In addition, χ^2^ test or Fisher’s exact test were used to compare the variations in CTC phenotype and disease status.

Receiver operating characteristic (ROC) curves were constructed to evaluate the performance of CTC phenotypes for predicting 1-year PFS rate. The area under each ROC curve (AUC) was calculated to assess the discriminating power, and Youden index (sensitivity + specificity − 1) was calculated to select the optimal cut-off values for CTC distribution. The Kaplan–Meier PFS curves were plotted to verify the cut-off criteria for prognosis evaluation based on EMT marker composition. Multivariate hazard ratios for PFS were estimated with Cox proportional hazards regression analysis.

The statistical analyses were conducted using SPSS software, version 22.0 (SPSS Inc., Chicago, IL, USA), and *P* values less than 0.05 were considered statistically significant. A nomogram was constructed to graphically visualize our predictive model according to the results of multivariable Cox regression analysis using the rms package in R version 3.4.1 (http://www.r-project.org/) [22]. The discriminative ability and accuracy of the nomogram were measured using concordance index (C-index) [23].

## Results

### Patient characteristics

In total, 108 women with HER2-negative metastatic breast cancer were enrolled between November 2013 and May 2017, and CTC phenotype analyses based on EMT marker composition were performed for each patient at least once. CTCs were detectable in the peripheral blood samples of 90 (83.3%) patients at baseline, and their clinicopathological characteristics are summarized in Table 1.

**Table 1:** Clinicopathological characteristics of 90 HER2-negative metastatic breast cancer patients with detectable CTCs in the peripheral blood samples at baseline

Characteristic	Number of patients (%)
Age (years)	
< 40	9 (10.0)
≥ 40 to < 60	64 (71.1)
≥ 60	17 (18.9)
Menstrual status	
Premenopausal	31 (34.4)
Menopausal	59 (65.6)
ER status	
Positive	71 (78.9)
Negative	19 (21.1)
PR status	
Positive	66 (73.3)
Negative	24 (26.7)
HR status	
Positive	74 (82.2)
Negative	16 (17.8)
Disease-free survival (months)	
≤ 12	18 (20.0)
> 12 to ≤ 24	9 (10.0)
> 24 to ≤ 60	34 (37.8)
> 60	29 (32.2)
Number of metastases	
Single	4 (4.4)
Multiple	86 (95.6)
Position of metastatic site	
Non-visceral	31 (34.4)
Visceral	59 (65.6)
Previous endocrinotherapy (after confirmed tumor relapse)	
None	71 (78.9)
First-line	15 (16.7)
Second-line or more	4 (4.4)

*CTCs* circulating tumor cells, *HER2* human epidermal growth factor receptor 2, *ER* estrogen receptor, *PR* progestrone receptor, *HR* hormone receptor

The median age of the 90 patients was 51 (range 32–73) years, and 31 (34.4%) of them were premenopausal. In addition, 74 (82.2%) patients were estrogen receptor- and/or progestrone receptor-positive, and 59 (65.6%) had visceral metastasis. Only 19 (21.1%) patients underwent first-line or further endocrinotherapy after confirmed tumor relapse.

### Association between CTC phenotype and clinicopathological characteristics

As depicted in Table 2, the average EMT-CTC count in 5 mL peripheral blood was almost two times higher in HR-negative patients than in HR-positive patients (15.19 ± 3.90 vs. 8.69 ± 1.42, *Z* = − 2.314, *P* = 0.021). However, total CTC count and other CTC phenotypes had no significant associations with HR status, DFS, and previous endocrinotherapy.

**Table 2:** Association between CTC phenotype and clinicopathological characteristics of patients with HER2-negative metastatic breast cancer

Characteristic	Total CTCs			E + CTCs			E +/M + CTCs			M + CTCs			EMT-CTCs		
	Average count	*Z* value	*P* value	Average count	*Z* value	*P* value	Average count	*Z* value	*P* value	Average count	*Z* value	*P* value	Average count	*Z* value	*P* value
HR status															
Positive	10.47 ± 1.56	− 1.942	0.052	1.93 ± 0.34	− 0.121	0.904	5.99 ± 1.07	− 1.687	0.092	2.70 ± 0.56	− 1.881	0.060	8.69 ± 1.42	− 2.314	0.021
Negative	17.06 ± 4.38			1.88 ± 0.61			9.00 ± 2.64			6.19 ± 2.33			15.19 ± 3.90		
Disease-free survival (months)															
< 24	15.04 ± 4.08	− 0.181	0.856	2.44 ± 0.64	− 1.148	0.251	7.81 ± 2.49	− 0.381	0.703	4.78 ± 1.49	− 0.963	0.335	12.59 ± 3.60	− 0.027	0.979
≥ 24	10.19 ± 1.26			1.70 ± 0.33			5.97 ± 0.95			2.70 ± 0.62			8.67 ± 1.20		
Previous endocrinotherapy															
None	11.73 ± 1.77	− 0.084	0.933	1.93 ± 0.35	− 0.557	0.578	6.41 ± 1.16	− 0.681	0.496	3.55 ± 0.77	− 0.355	0.722	9.96 ± 1.62	− 0.447	0.655
First-line or more	11.32 ± 2.83			1.89 ± 0.59			6.95 ± 1.92			2.47 ± 0.77			9.42 ± 2.45		

All data of cell count are expressed as mean ± standard error in 5 mL peripheral blood

*HR* hormone receptor, *DFS* disease-free survival, *CTCs* circulating tumor cells, *E + CTCs* epithelial CTCs, *E +/M + CTCs* biphenotypic epithelial/mesenchymal CTCs, *M + CTCs* mesenchymal CTCs, *EMT-CTCs* E +/M + and M + CTCs

### Association between CTC phenotype and PFS

**Fig. 1:**
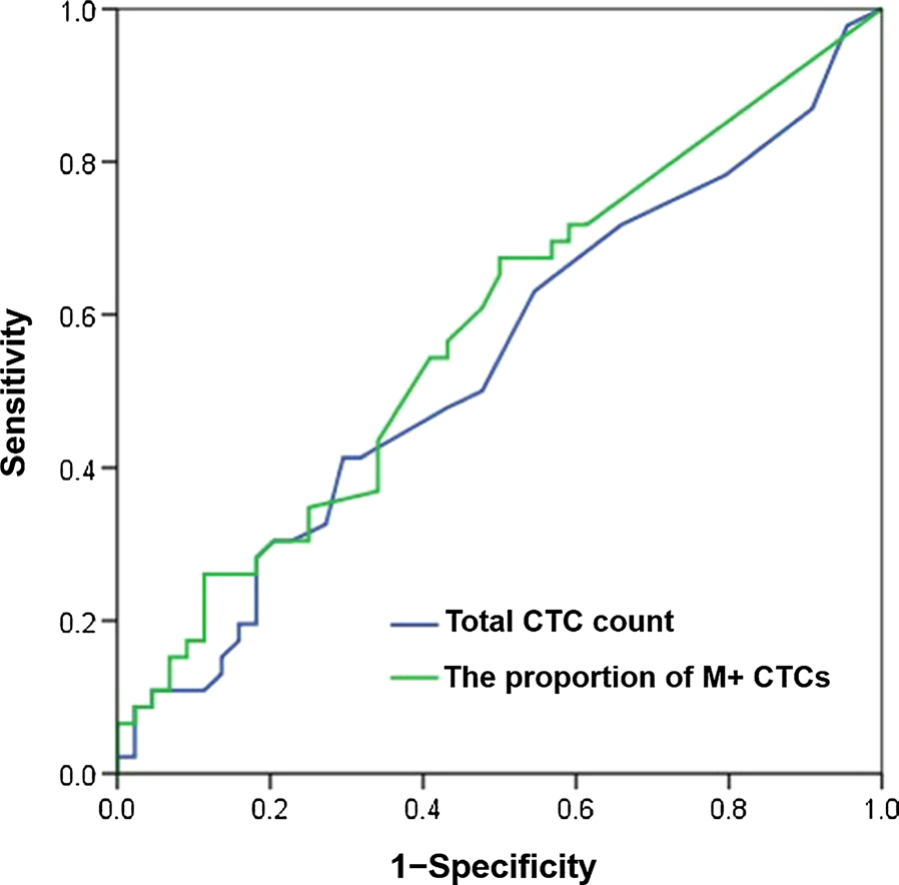
Receiver operating characteristic curves of total CTC count and the proportion of M + CTCs for predicting 1-year PFS rate. *CTC* circulating tumor cell, *M + CTCs* mesenchymal CTCs, *PFS* progression-free survival

The median PFS of all patients was 7.6 months (range 1.3–30.2 months). A total of 64 patients had disease progression during first-line chemotherapy, and peripheral blood samples were collected from 24 of them at the timepoint of disease progression. The ROC curve analyses of total CTC count and the proportion of M + CTCs for predicting 1-year PFS rate were performed, with respective AUCs of 0.538 (95% confidence interval [CI] = 0.418–0.657) and 0.581 (95% CI = 0.463–0.699) (Fig. 1). The cut-off value of total CTC count was 9.500, while that of the proportion of M + CTCs was 10.7%. Therefore, we proposed a total CTC count equals to or exceeds 10 per 5 mL peripheral blood with a proportion of M + CTCs surpassing 10.7% as the combined criteria for predicting short PFS.

**Fig. 2:**
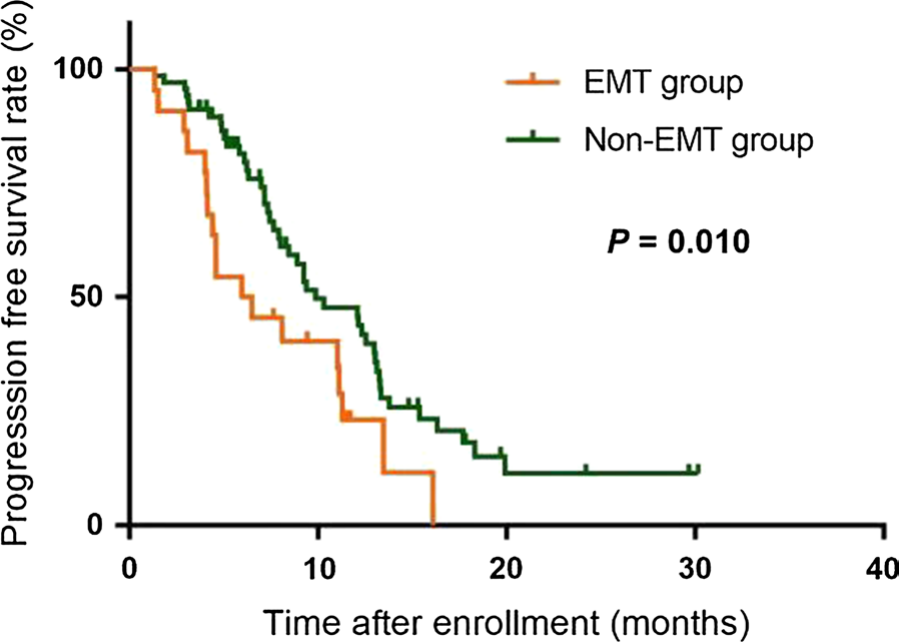
Kaplan-Meier analysis-based estimation of PFS probabilities of metastatic breast cancer patients in the EMT and non-EMT groups. Patients in the EMT group met the combined criteria with a total CTC count equaled to or exceeded 10 per 5 mL peripheral blood and a proportion of M + CTCs surpassed 10.7%; Patients in the non-EMT group did not meet the criteria. *PFS* progression-free survival

The patients who met the combined criteria were allocated into the EMT group, and those that did not match the criteria composed the non-EMT group. As depicted in Fig. 2, the median PFS was significantly shorter in the EMT group than in the non-EMT group (6.2 vs. 9.9 months, *P *= 0.010). Moreover, in the multivariate analysis, the combined criteria was an independent risk factor for PFS (Table 3).

**Table 3:** Multivariate analysis of clinicopathological characteristics and the combined criteria for predicting PFS

Variate	*P*	HR	95% CI
HR status	0.253	0.619	0.272–1.408
Previous endocrinotherapy	0.815	0.925	0.479–1.785
Position of metastatic site	0.172	0.654	0.356–1.203
Number of metastases	0.249	0.473	0.133–1.689
CA153	0.675	0.863	0.433–1.720
CEA	0.253	1.452	0.765–2.756
Combined criteria	0.003	2.688	1.407–5.134

*PFS* progression-free survival, *HR* hormone receptor, *CA153* carbohydrate antigen 153, *CEA* carcinoembryonic antigen, *HR* hazard ratio, *CI* confidence interval

**Fig. 3:**
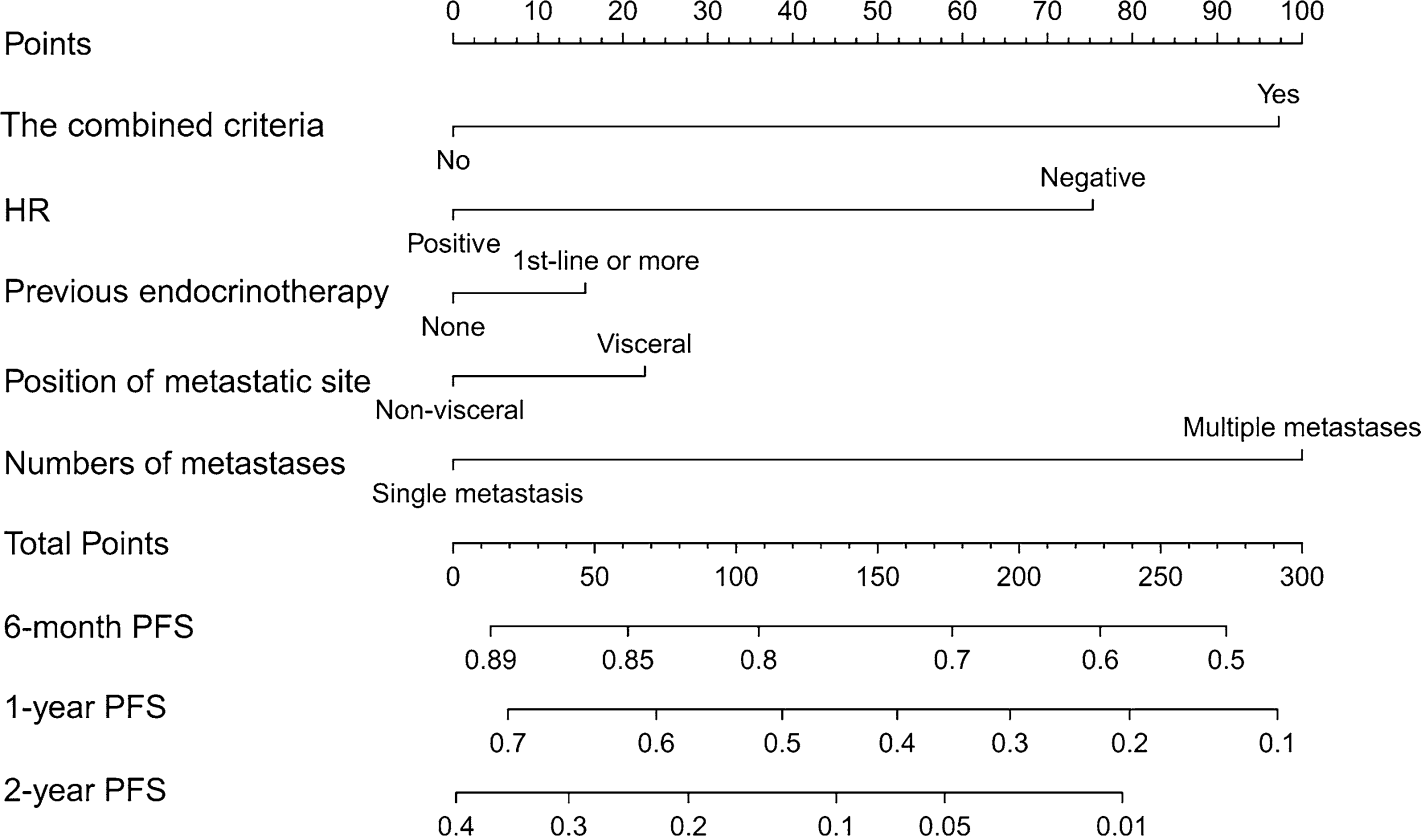
A prognostic nomogram for predicting PFS of patients with metastatic breast cancer. *PFS* progression-free survival, *HR* hormone receptor

According to the above analyses, a nomogram was constructed with certain clinicopathological characteristics (HR status, position of metastatic sites, number of metastases, and previous endocrinotherapy after confirmed tumor relapse) and the combined criteria to predict PFS (Fig. 3). The C-index of the nomogram for predicting PFS was 0.613 (95% CI = 0.527–0.699, *P *= 0.010).

### Association between CTC phenotype and disease status

When evaluating the distribution of CTC phenotypes based on EMT marker composition as a predictive index for tumor progression, increases in both total CTC count and the proportion of EMT-CTCs when compared with those measured after the prior therapeutic cycle should be considered. We compared the proportions of different CTC subpopulations between the time point of disease progression and the prior therapeutic cycle in the 24 patients with available peripheral blood samples and found that 5 had increased total CTC count, 7 manifested an increase in the proportion of EMT-CTCs, and 12 presented with both changes. As shown in Table 4, a significant difference in the variation pattern was observed between HR-positive and HR-negative groups (*χ*^2^ = 6.619*, P* = 0.037). Of the 5 patients who had an increase only in the total CTC count, 3 had triple-negative breast cancer. Comparatively, all of the 7 patients with an increase only in the proportion of EMT-CTCs had luminal-like breast cancer.

**Table 4:** Variation patterns of CTC phenotype in HR-positive and HR-negative groups

Increased index	HR-positive	HR-negative	*χ* ^2^	*P*
Total CTC count	2	3	6.619	0.037
The proportion of EMT-CTCs	7	0		
Both	10	2		

*HR* hormone receptor, *CTC* circulating tumor cell, *EMT* epithelial–mesenchymal transition

**Fig. 4:**
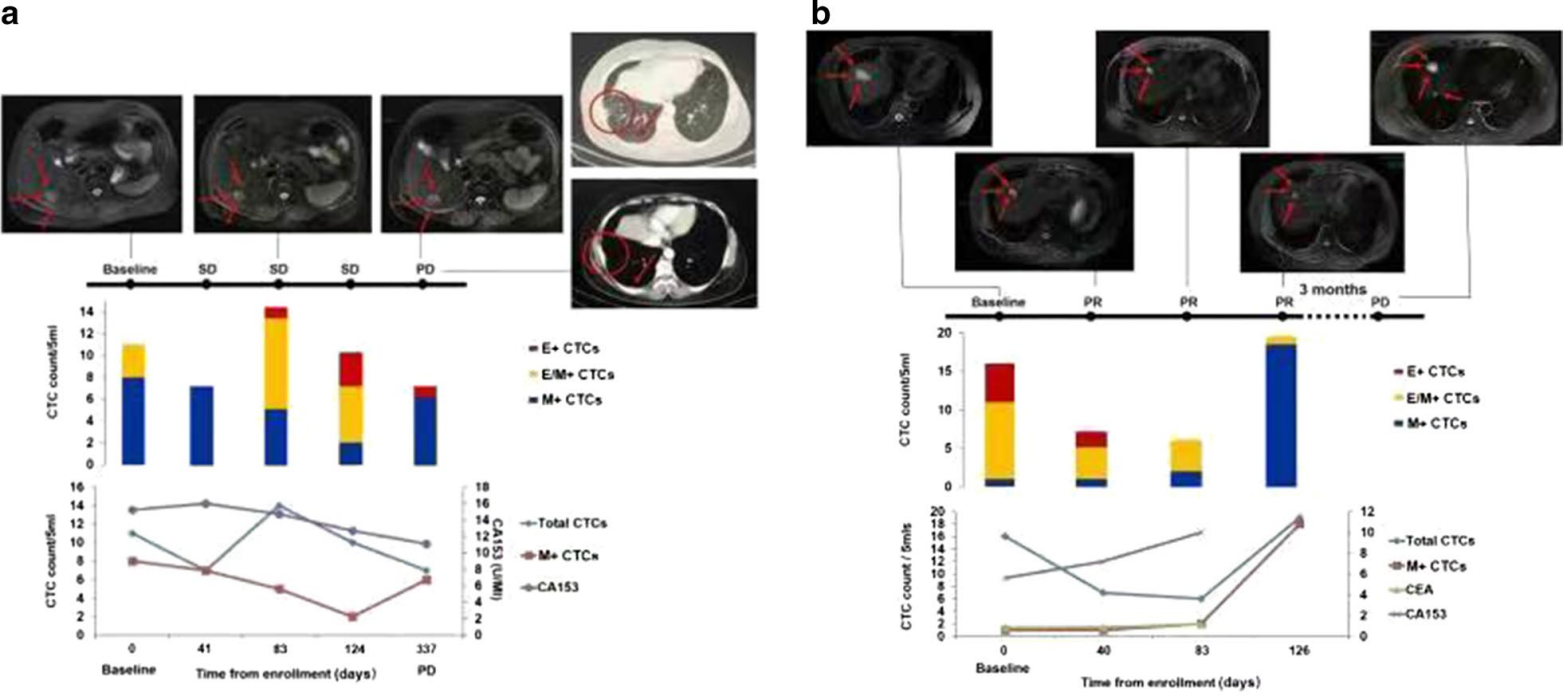
Comparison of total CTC count, CTC phenotype, serum tumor markers, and radiographic examination in identifying disease progression. **a** For patient No. 01058, the M + CTC count and their proportion in total CTCs manifested a continuous declining tendency and the total CTC count was fluctuated when the patient responded to chemotherapy. Moreover, when disease progression presented in the liver and pleura, the count and proportion of M + CTCs increased significantly compared with the prior measurement, while the total CTC count and the level of CA153 continued to descend. **b** For patient No. 01131, the total CTC count and M + CTC count were obviously increased 3 months earlier than the imaging evidence of disease progression. *CTC* circulating tumor cell; *E + CTCs* epithelial CTCs, *E +/M + CTCs* biphenotypic epithelial/mesenchymal CTCs, *M + CTCs* mesenchymal CTCs, *CA153* carbohydrate antigen 153, *CEA* carcinoembryonic antigen, *PR* partial response, *SD* stable disease, *PD* progressive disease. The arrows and circles on the radiographic image were used to indicate the lesions

The distribution of CTC subpopulations based on EMT markers was dynamically tracked and simultaneously compared with radiographic examination results and serum tumor marker levels. As depicted in Fig. 4a, for patient No. 01058, during chemotherapy with capecitabine and docetaxel, the total CTC count was fluctuated, while the M + CTC count and their proportion in total CTCs manifested a continuous declining tendency. Meanwhile, the patient exhibited radiographically stable disease according to the RECIST guidelines. Afterwards, the patient received maintenance chemotherapy with capecitabine of metronomic dosage. After 7 months of maintenance chemotherapy, the total CTC count and the level of CA153 continued to descend, while the count and proportion of M + CTCs increased significantly, along with development and progression of distant metastases in the liver and pleura. Furthermore, longitudinal tracing of the distribution of CTC phenotypes made it possible to monitor the disease status prior to imaging. Taking patient No. 01131 as an example (Fig. 4b), the total CTC count and M + CTC count were obviously increased 3 months earlier than the imaging evidence of disease progression.

## Discussion

In the present study, we investigated the prognostic value of dynamic CTC detection based on EMT markers during the first-line chemotherapy in patients with HER2-negative metastatic breast cancer and framed the criteria combining a total CTC count equals to or exceeds 10 per 5 mL peripheral blood and a proportion of M + CTCs surpasses 10.7%, which was significantly associated with prognosis and therapeutic response.

Previous metastatic breast cancer studies have suggested that the count and proportion of M + CTCs may be more appropriate for predicting therapeutic resistance and assessing prognosis than total CTC count [16, 17, 24]. In the present study, we took both the total CTC count and the proportion of M + CTCs into consideration to augment the accuracy of CTC detection in prognosis evaluation. In the multivariate analysis, the combined criteria regarding the heterogeneity of CTCs were demonstrated to be a significant predictor of the PFS of metastatic breast cancer patients before initiating new therapy. Additionally, we constructed a nomogram based on the criteria in combination with other significant clinicopathological characteristics to predict clinical outcomes of first-line chemotherapy in patients with HER2-negative metastatic breast cancer. Given that a previous study established nomograms according to the CTC count as a continuous covariate detected by the EpCAM-based CellSearch system allied with clinicopathological characteristics [25], it should be highlighted that our combined criteria model also assessed EMT marker composition, which was scarcely considered in previous studies, and thus, may serve as a tool to assist oncologists in tailoring proper regimens and controlling the dose intensity of chemotherapy.

Through longitudinal tracking of EMT features in CTCs, patients responding to the treatment were observed to manifest a declining tendency in total CTC count or an increasing proportion of E + CTCs, whereas those exhibiting disease progression presented with an increase in total CTC count or an increasing proportion of M + CTCs. Therefore, increases in both the total CTC count and the proportion of M + CTCs may be associated with short PFS and may predict disease progression. As demonstrated in the cases above, the proportion of M + CTCs is a more accurate index than total CTC count and serum tumor marker levels and may identify disease progression earlier than radiographic examination. Our observation was consistent with the findings by Yu et al. [16] that variations in the proportion of different CTC phenotypes accompanied response to each cycle of therapy and that disease progression and therapeutic resistance might be recognized in advance by monitoring CTC phenotype in metastatic breast cancer patients. Apart from breast cancer, some studies have also investigated the application of CTCs with EMT characteristics in therapeutic monitoring and prognosis evaluation for metastatic colon cancer [26] and lung cancer patients [27, 28], and these studies suggested the potential prognostic value of monitoring CTC phenotype based on EMT marker composition in clinical practice.

We also explored the association between CTC phenotype and clinicopathological characteristics. With regard to the HR status of primary tumors, the baseline average EMT-CTC count in patients with triple-negative breast cancer visibly exceeded that in HR-positive patients, whereas no significant difference was observed in total CTC count, which was in accordance with the finding of Yu et al. [16] that the CTC phenotypes in patients with luminal-like breast cancer were predominantly epithelial, whereas those in patients with triple-negative breast cancer were mostly mesenchymal. Moreover, the variation pattern of CTC phenotypes between the time point of imageologically observed disease progression and the prior therapeutic cycle was different between luminal-like breast cancer and triple-negative breast cancer patients, which may due to the distribution of different CTC phenotypes associated with HR status. With disease progression during treatment, the EMT process of E + CTCs may be easier to observe in luminal-like breast cancer patients with predominantly E + CTCs, whereas triple-negative breast cancer patients with mostly M + CTCs may present with ascending total CTC count.

As suggested in the present study, constructing criteria that take both the total CTC count and the proportion of CTC subpopulations into consideration and framing the corresponding nomogram system in this straightforward and practical detection approach may contribute to the improvement of prognostic evaluation and tailoring of individual treatment decisions. However, the statistical power of the present study was limited due to the small sample size, and thus, further large-scale prospective trials containing overall survival data are needed to verify the prognostic criteria model.

## Conclusions

In conclusion, the combined criteria that considered both the total CTC count and the proportion of M + CTCs was observed to be significantly associated with prognosis and presented sensitivity superior to that of simple total CTC count in prognostic estimation and therapeutic response monitoring for patients with HER2-negative metastatic breast cancer. Moreover, CTC phenotype exhibited a great dynamic variation trend that associated with changes in therapeutic response. This straightforward and practical CTC detection approach based on EMT markers could allow dynamic monitoring of therapeutic response and may considerably contribute to personalization of metastatic breast cancer treatment.

## Abbreviations

EMT: epithelial–mesenchymal transition
CTCs: circulating tumor cells
EpCAM: epithelial cell adhesion molecule
CI: confidence interval
FDA: Food and Drug Administration
E + CTCs: epithelial CTCs
CTCs E+/M + CTCs: biphenotypic epithelial/mesenchymal
M + CTCs: mesenchymal CTCs
HR: hormone receptor
RECIST guidelines: the Response Evaluation Criteria in Solid Tumors guidelines
PFS: progression-free survival
ROC curves: receiver operating characteristic curves
AUC: area under the curve
AUC-ROC: the area under each ROC curve
DFS: disease-free survival
CA153: carbohydrate antigen 153
CEA: carcinoembryonic antigen
PR: partial response
SD: stable disease
PD: progressive disease
RNA-ISH: RNA-in situ hybridization
bDNA: branched DNA
